# Control of Browning, Enzyme Activity, and Quality in Stored Fresh-cut Fruit Salads through Chitosan Coating Enriched with Bergamot Juice Powder

**DOI:** 10.3390/foods13010147

**Published:** 2024-01-01

**Authors:** Bahar Demircan, Yakup Sedat Velioglu

**Affiliations:** Department of Food Engineering, Ankara University, 06850 Golbasi, Ankara, Turkey; bdemircan@ankara.edu.tr

**Keywords:** fresh cut, fruit salad preservation, edible coating technology, bergamot juice powder extract, extended shelf life, quality improvement, enzymatic browning inhibition, polyphenol oxidase activity, microbial safety, sensory analysis

## Abstract

In this study, fresh-cut fruit salads composed of apples, pears, kiwis, and pineapples were stored at +4 °C for 18 days under distinct conditions: non-coated (NC), chitosan-coated (CH), and bergamot juice powder extract-enriched chitosan-coated (CHBE). Storage endpoint decay percentages were as follows: NC group: 100%, CH group: 26.67–53.3%, CHBE group: 13.33–26.67%. CHBE had the highest moisture content (87.05–89.64%), soluble solids (12.40–13.26%), and chroma values (2.35–6.60). CHBE and NC groups had 2.10% and 6.61% weight loss, respectively. The NC group had the highest polyphenol oxidase activity (19.48 U mL^−1^) and browning index (0.70 A_420_/g); CH group: 0.85 U mL^−1^, 0.35 A_420_/g; CHBE group: 0.57 U mL^−1^, 0.27 A_420_/g. CHBE showed a titratable acidity of 1.33% and pH 3.73 post-storage, impeding microbial proliferation with the lowest counts (2.30–3.24 log CFU g^−1^). The microbial suitability of the NC group diminished after day 6, with an overall preference score of 1.00. Conversely, the CH and CHBE groups scored 3.15 and 4.56, highlighting the coatings’ effectiveness. Bergamot juice powder extract further enhanced this, mitigating browning and enhancing quality. Results reveal tailored coatings’ potential to extend shelf life, improve quality, and enhance fruit salads’ acceptability. This study underscores the importance of edible coatings in addressing preservation challenges, emphasizing their role in enhancing food quality and consumer acceptability. Incorporating edible coatings is pivotal in mitigating deterioration issues and ensuring the overall success of fresh-cut fruit products in the market.

## 1. Introduction

Fruit is crucial in human nutrition, offering significant health advantages [1]. They are rich in essential nutrients, including energy-providing compounds, fiber, vitamins, minerals, flavonoids, and phytochemicals, making them a cornerstone of a nutritious diet. The importance of fruit consumption for health is emphasized, and the growing demand for fruit has led to an increased focus on production and quality control. While fruit production is rising globally, the popularity of consuming fresh-cut fruit is also increasing [2]. However, challenges such as preserving shelf life persist, necessitating innovative solutions to maintain quality and reduce losses [3].

To extend the shelf life of fresh-cut fruit and enhance their quality, various preservation techniques are employed, addressing factors such as moisture loss, ripening, and microbial growth [4]. Among these methods, crucial roles are played by edible coatings and technologies like modified atmosphere packaging, low-temperature storage, high pressure, ozone, and pulsed light [5,6]. Edible coatings, which involve applying diverse materials onto fruit surfaces to limit gas exchange, offer a sustainable solution by improving shelf life and quality while reducing reliance on plastic packaging [2]. Integrating bioactive compounds into these coatings presents a promising avenue for enhancing nutritional and health benefits. These compounds positively affect health and can be harnessed to further elevate fresh-cut fruit’s nutritional content [1]. Notably, antioxidants found in bioactive compounds combat cellular damage and oxidative stress, and their inclusion in coatings could significantly boost antioxidant intake for consumers. Furthermore, incorporating vitamins, minerals, and fiber through coatings contributes to a more well-rounded and nutritious diet [6]. This convergence of edible coatings and bioactive compounds extends shelf life and enhances consumers’ overall health and well-being.

Consumer awareness has sparked an interest in natural methods of microbial control, leading to the incorporation of natural preservatives into enrichment-focused edible coatings [7]. While plant extracts have been utilized in biopolymer matrices, research on bergamot fruit extract remains limited, opening new avenues in this domain. Bergamot fruit, primarily used for essential oil production from its peel, holds untapped potential due to its bitter juice, which raises concerns about cost and environmental waste. However, bergamot juice contains valuable phenolic compounds with diverse functional benefits, positioning natural phenols in fruit juice as potential antioxidants and antimicrobial agents in the food industry. The predominant flavanone compound in bergamot juice, naringin, contributes to bitterness and is susceptible to high temperatures [8]. Through the dehydration of bergamot juice, bitterness can be eliminated while preserving its nutrient content, presenting an opportunity for its utilization as a natural antioxidant and antimicrobial component.

In this study, bergamot juice powder obtained by spray-drying waste bergamot juices as a source of phenolic components was employed to enhance chitosan coatings. The impact of these coatings on the shelf life of fresh-cut fruit salad was extensively evaluated through physical, chemical, sensory, and microbiological analyses. Utilizing bergamot juice as a protective element in coatings is a pioneering aspect of this research. Data analysis underscores the significant and innovative repurposing of waste streams for bioactive components. This study represents an original advancement in expanding the potential of edible coatings for food preservation, promoting sustainable resource use, and encouraging eco-conscious practices.

## 2. Materials and Methods

### 2.1. Material

The selected fresh-cut fruit salad, comprising green apples, pears, kiwis, and pineapples, was chosen based on seasonal availability and purchased from a local market in Ankara, Turkey. Each fruit was ensured to be fresh, undamaged, free of decay, and approximately the same weight (average fruit weights for one green apple, pear, kiwi, and pineapple were 170 g, 230 g, 125 g, and 1.6 kg, respectively) and size. After thorough washing, the fruit underwent sanitation by soaking in 5 ± 1 °C drinking water containing 200 mg L^−1^ active chlorine for 20 min, followed by rinsing with a 20 mg L^−1^ chlorine solution for 5 min. Using stainless-steel knives, peelers, and fruit–vegetable corers, the fruit was peeled and sliced to approximately 4 cm in diameter and 2 cm in thickness, resulting in pieces weighing approximately 3.5 ± 0.25 g each. Following the procedure outlined by Santana Moreira et al. [9], equal numbers of fruit salads (10 pieces each) were prepared for each fruit type.

The edible coating formulation used low-molecular-weight chitosan (EC 223-311-2, CAS 9012-76-4, Sigma-Aldrich, Co., Saint Louis, MO, USA). To enhance the coating formulation, readily available bergamot (*Citrus bergamia* Risso et Poiteau) fruit juice powder was previously produced in another study [8]. Analytical and chromatographically pure chemicals were sourced from reputable suppliers, including Sigma-Aldrich, Merck, Carlo Erba, Isolab, Riedel-de Haën, and Oxoid. Laboratory procedures and analyses were conducted using distilled water (Simsek Laborteknik, SS-204, Ankara, Turkey). All analyses were performed with three repetitions and in triplicate.

### 2.2. Preparation of Extract from Bergamot Juice Powder

Continuing our previous work [8], a 50% acetone (ACTN) solvent concentration produced a phenolic-rich bergamot juice powder extract. Initially, 1 g of powder was combined with ACTN to match the juice’s initial water-soluble solids content (7.93%). The mixture was vortexed (Heidolph, D-91126, Schwabach, Germany), transferred to capped glass tubes, and subjected to a 30 min ultrasound-assisted extraction (ISOLAB Laborgeräte, GmbH, Istanbul, Turkey) at 53 kHz frequency, 100% power, and 20 °C, following the parameters established in our preliminary trials. Post-extraction centrifugation (Hettich Zentrifugen Universal 320R, Tuttlingen, Germany) was carried out at 3354 g and 20 °C for 10 min. After removing the solvent using a rotary evaporator (Buchi R-114, Flawil, Switzerland), the residue was recovered with distilled water and stored in amber vials at −20 °C until ready.

### 2.3. Edible Coating Application to Fresh-Cut Fruit Salad and Storage Analysis

The edible coating solution was prepared by dissolving 2% (*w*/*v*) chitosan in a 1% (*v*/*v*) acetic acid solution at 25 °C with magnetic stirring (Heidolph, MR HeiStandard, Schwabach, Germany) at 500 rpm for 4 h, followed by 5 min homogenization at 13500 rpm using an Ultra-Turrax (Heidolph Instruments GmbH&Co., Silent Crusher M, Schwabach, Germany) with the addition of 3% (*v*/*v*) ACTN extract and 1.0% (*w*/*v*) glycerol [10]. For coating application, 140 g of fruit salad was dipped in the edible coating solution (100 mL) for 30 min. After the excess solution had been drained away using a draining rack for 10 min, the coated fruit was dried for an additional 10 min at room temperature. The coated (CH: pure chitosan and CHBE: chitosan enriched with bergamot juice powder extract) and non-coated (NC) fruit salads were stored in sealed polypropylene containers at +4 °C for 18 days in a laboratory-type fridge (UGUR USS 748 D2KL, Ankara, Turkey).

Individual analyses were conducted for decay percentage, moisture content, total soluble solid content, and fruit color. For pH, titratable acidity, browning index, enzyme activity, and microbial quality analyses, a mixture of fruit salads totaling approximately 15 g, with one piece from each fruit, was used. Weight loss and sensory evaluation analyses were performed on the entire fruit salad.

#### 2.3.1. Decay Percentage

The decay percentages of individual fruits were determined following the procedure outlined by Hashemi and Jafarpour [11]. Calculations were based on the numbers of the same type of fruit present in each fruit salad, utilizing Equation (1).

Decay (%) = (1n_1_ + 2n_2_ + 3n_3_) · 100/(3n) (1)

In Equation (1), n_1_, n_2_, and n_3_ represent the number of samples with different degrees of decay, and n represents the total number of tested samples. Here, a 4-level scale was employed, where 0 indicates no decay, 1 signifies slight decay, 2 represents moderate decay, and 3 indicates severe decay.

#### 2.3.2. Moisture Content

The moisture content (%) of individual fruits was determined gravimetrically by drying them in an oven (Simsek Laborteknik, Ankara, Turkey) at 105 °C until reaching a constant weight.

#### 2.3.3. Total Soluble Solid Content

The total soluble solid content (%) of individual fruits was directly determined using a digital refractometer (Atago, RX-5000, Saitama, Japan) at 25 °C [9].

#### 2.3.4. Color

The surface colors of individual fruits were determined by directly reading the L*, a*, and b* coordinates using a colorimeter (Konica Minolta, CR-400, Tokyo, Japan). Here, L* (Luminance/Brightness) indicates brightness on a scale of 0 to 100, with higher values indicating greater brightness. a* (Redness/Greenness) represents the red/green spectrum (negative/positive), while b* (Yellowness/Blueness) signifies the yellow/blue spectrum (negative/positive). The whiteness index (WI*), total color difference (ΔE*), chroma (ΔC*), and color index (CI*) have been calculated according to Equations (2)–(5), respectively, as reported by Fai et al. [12].

WI* = 100 − [(100 − L*)^2^ + a*^2^ + b*^2^]^1/2^ (2)

ΔE* = [(Δa*)^2^ + (Δb*)^2^ + (ΔL*)^2^]^1/2^ (3)

ΔC* = [(Δa*)^2^ + (Δb*)^2^]^1/2^ (4)

CI* = (a* · 1000)/(L* · b*) (5)

Before determining the color coordinates, the colorimeter was calibrated with a white calibration plate (Y = 93.7, X = 0.3121, y = 0.3195). In color difference calculations, the color coordinates of fruits in the control group were used as reference values.

#### 2.3.5. Titratable Acidity and pH

A 15 g fruit salad was homogenized in 50 mL of distilled water using a blender, followed by filtration through coarse filter paper to produce the fruit salad homogenate. The pH of the obtained clear liquid was determined at room temperature using a pH meter (WTW, InoLab 720, Munich, Germany). The acidity (%), in terms of citric acid, was determined by titrating 5 mL of the homogenate (15 g sample/50 mL distilled water) with 0.1 M sodium hydroxide solution until the color turned pink in the presence of phenolphthalein indicator [9].

#### 2.3.6. Browning Index

The browning index (A_420_/g sample) was determined following the method established by Sobral et al. [13]. The fruit salad homogenate was used, and the absorbance at 420 nm was directly measured using a UV–VIS spectrophotometer (Shimadzu, UV-VIS 1601, Tokyo, Japan).

#### 2.3.7. Extraction and Analysis of Polyphenol Oxidase Enzyme Activity

A crude extract solution was prepared using the fruit salad homogenate as the base for enzymatic oxidation studies. A mixture of 2 mL distilled water, 100 µL substrate solution (0.5 M catechol solution), and 500 µL crude extract solution was combined for the fruit salad sample. The blank sample consisted of 2 mL distilled water and 500 µL substrate solution. Absorbance was measured at 420 nm using a UV–VIS spectrophotometer, recording changes every 30 s for 3 min. Enzyme activity was calculated as 0.001 units of absorbance change per min per 1 mL of enzyme solution, determined from the slope of the initial linear segment of the absorbance–time curve. Calculations followed Equation (6) in Karagoz and Demirdoven [14].

Activity (Unit mL^−1^) = (E/0.001) · (1/H_e_) · H_rk_ · S_f_) (6)

Here, E represents the slope of the initial linear portion of the absorbance–time curve (Δabsorbance min^−1^). The constant value 0.001 converts the absorbance min^−1^ value to units. H_e_ stands for the volume (mL) of the enzyme extract within the reaction mixture, H_rk_ indicates the total volume (mL) of the reaction mixture, and S_f_ represents the dilution factor.

#### 2.3.8. Determination of Microbial Quality

Under aseptic conditions, 10 g of fruit salad was homogenized with 90 mL of sterile 0.85% physiological saline solution to establish decimal dilutions using the same diluent. After initial experimentation, three suitable serial dilutions were chosen, and the spread plate technique was employed to inoculate the respective culture media, with incubation periods per specific requirements. Total mesophilic bacteria (TMAB) was enumerated at 28 °C for 48 h using Plate Count Agar (PCA, Rahway, NJ, USA, Merck). Total psychrotrophic bacteria (TPB) enumeration took place at 4 °C for 10 days using PCA. Total yeast and mold (TYM) was generated at 28 °C for 3–5 days using Yeast Glucose Agar (YGC, Merck). Finally, total *Enterobacteriaceae* (TEN) enumeration was performed at 37 °C for 24 h using Violet Red Bile Glucose Agar (VRBG, Merck). The microbial load was expressed as log colony-forming units (CFU) per gram of fruit salad [15].

#### 2.3.9. Weight Loss

The weight loss (%) in fruit salads was determined by calculating the percentage change from the initial fresh weight for each storage period [12].

#### 2.3.10. Sensory Evaluation

A panel of 15 semi-trained assessors from the Department of Food Engineering at Ankara University conducted sensory evaluations of fruit salads in the sensory analysis laboratory throughout the designated storage periods. The sensory assessment included non-coated fruit and fruit salads coated with two distinct formulations. These samples were assigned random three-digit, non-consecutive codes and presented to the assessors in a randomized sequence. The sensory analysis followed the methodology outlined by Lacivita et al. [15], utilizing a scoring system ranging from 1 to 5 for attributes such as odor, appearance, flavor, texture, and overall preference. The scale ranged as follows: 1 = very bad; 2 = bad; 3 = neither bad nor good; 4 = good; and 5 = very good. The evaluation covered the entire fruit salad, with panelists instructed to assign scores to each attribute individually and to use the same scale to assess the overall quality of each fruit salad sample. The analyses were conducted in the sensory analysis laboratory.

### 2.4. Statistical Analysis

The collected data underwent factorial analysis of variance (ANOVA) followed by Duncan’s test for comparison. All statistical analyses were conducted using SPSS 22.0 software (SPSS Inc., Chicago, IL, USA), with significant differences at *p* < 0.05.

## 3. Results

**Figure 1:**
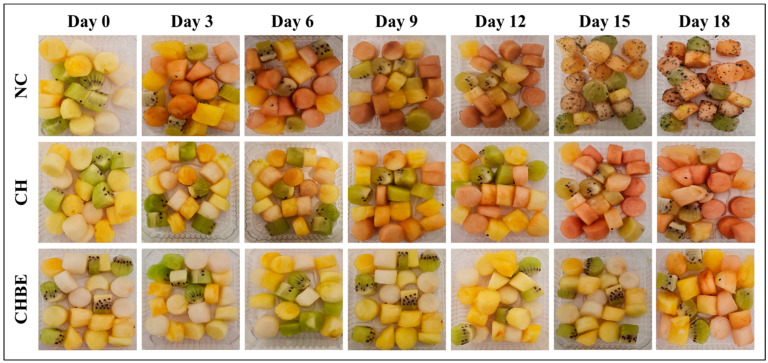
The visual impact of different coatings on the appearance of fruit salads during storage (NC: non-coated; CH: chitosan-coated; CHBE: bergamot juice powder extract-enriched chitosan-coated).

The images of the NC-, CH-, and CHBE-group fruit salads were captured at 3-day intervals over an 18-day storage period, as shown in Figure 1. As observed, there are noticeable differences among the sample groups throughout the storage period. While visual deterioration is evident from the third day in the NC group, it becomes apparent only on the ninth day in the CH group and on the eighteenth day in the CHBE group. The most influential factor affecting visual quality is the browning of the white-fleshed fruits, which is particularly pronounced in the images. In addition to visual degradation in the NC group, microbiological spoilage is also significantly pronounced from the fifteenth day onwards, with no similar appearance in the other sample groups.

### 3.1. Decay Percentage

The decay percentage analysis monitors fruit deterioration and shelf life by observing symptoms such as softening, discoloration, mold growth, and odor [16]. Initial decay percentages were set at 0% for all fruit samples at the start of storage. Throughout the storage period, rapid decay was observed in all fruits of the NC group (Table 1). This aligns with findings from Hashemi and Jafarpour [11], who reported faster decay in a control group of kiwis compared to those with coatings. Decay symptoms were observed in the NC group from the third day. In the CH and CHBE groups, decay symptoms started on the third day for apples and pears, the ninth day for kiwis, and the twelfth day for pineapples.

The processing of fresh-cut fruits accelerates metabolic reactions, leading to tissue damage, stress, and higher decay percentages in the control group. Edible coatings significantly reduced decay percentages. By the end of storage, while the NC group exhibited 100% decay, the CH and CHBE groups showed reduced decay percentages. For apples, pears, pineapples, and kiwis, decay percentages were 53.33%, 46.67%, 26.67%, and 40.00% in the CH group and 33.33%, 26.67%, 13.33%, and 20.00% in the CHBE group, respectively. Overall, apples were the least resistant to decay, while pineapples demonstrated the highest resilience. These results align with previous research on decay indices in fresh-cut papayas [17], reduced mold growth on coated strawberries [18], and decay reduction in strawberries through chitosan coatings [19]. Similar effects have been observed in mangoes and guavas [20,21], pineapple decay rates [16], and extended pear shelf life with specific coatings [22].

This study concludes that CH and CHBE coatings effectively reduce fresh-cut fruit decay percentages, extending shelf life by creating a protective barrier against water loss, regulating gas exchange, and inhibiting microbial contamination. The findings support the decay-reducing properties of CH coatings and the enhanced efficacy of CHBE coatings enriched with bergamot extract. The interaction between bergamot extract and chitosan may contribute to these effects, similar to coatings enriched with papaya leaf extract and higher polyphenol concentrations [23]. Decay percentage is a critical indicator of fruit quality, shelf life, and potential storage, transportation, or disease concerns, emphasizing the importance of preservation optimization for industry success. This study underscores the effectiveness of CH and CHBE coatings in mitigating decay across various fresh-cut fruits.

### 3.2. Moisture Content

Moisture content is a critical determinant of fresh-cut fruit quality, texture, and shelf life, assessable through weight or specialized moisture measurement devices. Shifts in moisture content during storage are closely monitored, with visible cues like wrinkling indicating reduced moisture levels. Our results revealed moisture loss across all sample groups during storage (Table 1). Inherent damage from processing, such as cutting or peeling, can compromise fruit structure and contribute to water loss [5]. Notably, the NC group displayed the highest moisture loss throughout storage: apples (10.74%), pears (9.84%), pineapples (8.38%), and kiwis (8.97%). In contrast, the CH group exhibited reduced moisture loss: apples (2.93%), pears (2.46%), pineapples (2.01%), and kiwis (2.33%); similarly, the CHBE group showcased moisture loss percentages: apples (2.39%), pears (2.25%), pineapples (1.78%), and kiwis (2.01%). Edible coatings establish semipermeable barriers, impeding water migration and substance movement [4,24], a phenomenon evidenced across diverse fruit [25].

Pears exhibited the highest moisture loss among the studied fruit, while pineapples showed the least. Initial moisture levels on the first day were as follows: apples (84.58%), pears (86.72%), pineapples (88.00%), and kiwis (87.10%) for the NC group; apples (88.75%), pears (86.72%), pineapples (90.93%), and kiwis (90.45%) for the CH group; and apples (89.18%), pears (90.05%), pineapples (91.26%), and kiwis (90.49%) for the CHBE group. Correspondingly, on the final storage day, levels were as follows: apples (75.50%), pears (78.19%), pineapples (80.63%), and kiwis (79.29%) for the NC group; apples (86.15%), pears (87.81%), pineapples (89.10%), and kiwis (88.34%) for the CH group; and apples (87.05%), pears (88.02%), pineapples (89.64%), and kiwis (88.67%) for the CHBE group, possibly influenced by the coating method. Chitosan-based coatings, known for moisture retention capabilities [4], effectively counter moisture loss, aligning with findings from Donsì et al. [26] and Silva et al. [27] that demonstrated chitosan’s efficacy in reducing water loss in various fruit. Our study underscores the direct link between rapid moisture loss and heightened decay percentages, emphasizing the role of moisture in decay prevention.

The exceptional moisture preservation of the CHBE coating can be attributed to phenolic compound–chitosan interactions leading to cross-linking facilitated by antioxidants from bergamot juice powder. This interaction prevents cellular damage and enhances water retention. Meanwhile, the CH coating is a barrier against water vapor, with bergamot extract acting as an anti-transpirant and antioxidant [28]. Edible coatings are crucial in slowing water vapor and transpiration, preserving freshness, tissue integrity, and shelf life by reducing permeability, inhibiting air penetration, and minimizing moisture evaporation, as observed in weight changes [29]. This study reaffirms the effectiveness of CH and CHBE coatings in countering moisture loss in fresh-cut fruit.

### 3.3. Total Soluble Solid Content

Fresh-cut fruit’s total soluble solid content, including sugars, acids, vitamin C, and specific pectins, serves as a sweetness indicator based on dissolved sugar concentration, influencing consumer preference. Storage impacts on soluble solids involve (i) ripening, leading to increased sugar content and values; (ii) respiration depleting sugars and decreasing total soluble solids; and (iii) water loss concentrating sugars and elevating total soluble solids. Generally, total soluble solids rise during storage [29].

In the NC group, initial total soluble solids decreased until day 6 due to respiration, then increased due to water loss. Coated samples displayed lower initial total soluble solids, progressively increasing during storage (Table 1).

The CH and CHBE groups initially had lower total soluble solids compared to the NC group: CH group values were 12.55% (apples), 12.04% (pears), 11.27% (pineapples), and 11.41% (kiwis); CHBE group values were 12.03%, 11.49%, 10.81%, and 11.03%. By the end of storage, the CH group reached 14.03%, 13.39%, 12.51%, and 12.68%, while the CHBE group reached 13.37%, 12.73%, 11.84%, and 12.60%. The literature supports lower total soluble solids in coated fruit, with studies revealing higher soluble solids in untreated mangoes than in chitosan-coated ones [30]. Souza et al. [31] attributed reduced soluble solids in coated fruit to vapor barrier effects, slowing metabolic activity and solid degradation.

Coatings may have preserved total soluble solids by serving as a barrier against gases that break down sugars, such as oxygen and ethylene, or by preventing the contamination of microorganisms that consume sugars on the fruit surface. Post-storage, total soluble solids increased for apples (16.80%), pears (16.43%), pineapples (15.05%), and kiwis (15.60%) in the NC group; 11.79%, 11.21%, 11.00%, and 11.13% in CH; and 11.42%, 11.18%, 10.45%, and 10.53% in CHBE. CHBE had the lowest increases due to bergamot extract’s antioxidant properties inhibiting enzymatic activities, particularly invertase, preserving sugar and total soluble solids. CH maintained sugar concentration, while CHBE’s added antioxidant and enzyme inhibition properties maintained the stability of total soluble solids. This study underscores the effectiveness of CH and CHBE coatings in preserving total soluble solids in diverse fresh-cut fruit.

### 3.4. Color

Color is a crucial quality indicator for consumers, influencing fruit attractiveness and purchase decisions. Storage induced significant shifts in fruit groups’ L*, a*, and b* values (Table S1).

The NC group experienced reductions in L* from −15.89% to −21.58%, the CH group showed a −9.85% to −11.48% reduction, and the CHBE group displayed a −8.62% to −10.21% reduction. Apples exhibited the most pronounced change in L*, while pineapples showed the slightest alteration. Coatings effectively preserved higher L* values, acting as barriers against oxidation and color modification.

Coated fruit consistently maintained higher a* values over storage, with milder decreases (−30.74% to −41.91% and −26.10%) compared to the significant negative changes observed in the NC group (−40.73% to −51.53%). Pears exhibited positive a* values, preventing a reduction in greenness, while apples displayed increased redness (+a*). Changes in +b* values varied among fruit groups during storage, with increases observed in apples, pears, and kiwis, while pineapples showed a decrease. At the end of the storage period, +b* value changes for the NC group were as follows: apples (+25.04%), pears (+70.24%), kiwis (+17.68%), and pineapples (−34.44%); for the CH group: apples (+13.63%), pears (+62.05%), kiwis (+15.31%), and pineapples (−31.85%); and the CHBE group: apples (+13.01%), pears (+53.16%), kiwis (+10.14%), and pineapples (−29.31%). Coated fruit consistently displayed higher +b* values, intensifying yellowness in apples, pears, and kiwis while mitigating it in pineapples. These color attribute variations correlate closely with natural changes and deterioration indicators.

Adding bergamot juice powder extract to the coating formulation showcased heightened efficacy in mitigating color alterations, attributed to its antioxidative and anti-inflammatory attributes that effectively slowed down oxidative and inflammatory processes. This contribution likely stabilized the vulnerable natural pigments, preserving the vibrant colors. Notably, the CHBE coating enriched with bergamot juice powder extract demonstrated superior color stability, underpinned by its antioxidative, pigment-stabilizing, and anti-inflammatory qualities.

These observed color changes intertwine with fruit moisture loss and total soluble solids values, where moisture reduction directly influences color shifts, often leading to darker shades and decreased L* values. In contrast, elevated total soluble solids content enhances color intensity, especially for red or yellow hues. The color data of the fruit produced calculated WI*, ΔE*, ΔC*, and CI* values (Table S2). WI* is related to fruit lightness, which generally increases as fruit pales during storage. ΔE* denotes the color difference between measured (CH and CHBE groups) and reference (NC group) colors, with apples and pears experiencing color loss during storage, yielding higher ΔE* values. ΔC* reflects color saturation, which typically decreases over time, with pineapples and kiwis displaying reduced color vibrancy, resulting in lower ΔC* values. CI* indicates color intensity, diminishing due to fading or darkening throughout storage.

These attributes are crucial for assessing fruit quality, freshness, and marketability, serving as visual consumer indicators that influence marketing and quality control. Color changes during storage significantly impact shelf life and consumer perception. Moreover, these changes may signify enzymatic and chemical reactions, affecting nutritional content and edibility. Coatings effectively minimized color changes, resulting in lower ΔE* values or consistent coloration. They act as protective barriers, preserving color and sustaining higher ΔC* values, contributing to smoother and shinier surfaces, and enhancing brightness and whiteness throughout storage. Numerous studies have explored the effects of edible coatings on the color, opacity, and glossiness of fresh-cut fruit, aligning with the findings of this study [32]. Coatings containing antioxidants, such as Irish moss and whey protein, effectively controlled or reduced color changes in apples, pears, and papayas [28,33]. Alginate and gellan coatings enriched with antioxidants upheld visual quality in carrots, jicama, and mangoes [6].

### 3.5. Browning Index

The browning index, a measure of enzymatic browning resulting from the interaction between phenolic compounds and enzymes causing brown color formation, is quantified through absorbance and reflects pigment intensity. This index is essential for evaluating fresh-cut fruit quality, shelf life, and consumer preference.

A lower index sustains visual appeal, prevents undesired color changes, and provides insights into phenolic compounds and nutritional value. Initial variations in the browning index were observed across groups, with the NC group exhibiting the highest value (0.38 A_420_/g) and the CHBE group exhibiting the lowest (0.19 A_420_/g).

Coated fruit salads exhibited enhanced glossiness due to the coating layer, resulting in lighter-colored homogenates and lower browning indices. The choice of polymers and additives within the coating matrix could have influenced coloration. At the same time, the CHBE group had the lowest index due to the whitish–yellowish hue of the bergamot juice powder in the coating, potentially affecting homogenization color. Browning indices consistently increased throughout storage, with the most significant rise observed in the NC group and the least in the CHBE group (Table 2).

By the end of storage, the browning index had increased by 82.89% in the NC group, 60.91% in the CH group, and 42.10% in the CHBE group. The lack of coating exposed fruit salads in the NC group to air, accelerating oxidation and resulting in the highest browning index (0.70 A_420_/g). A correlation between heightened browning index and moisture loss in individual fruit during storage suggested compound concentration and increased enzymatic browning, aligning with changes in fruit color values driven by moisture loss. Rojas-Grau et al. [33] established a parameter for monitoring enzymatic browning, indicating that an elevated a* value corresponds to an increased browning index during apple storage.

At the end of storage, the browning index displayed significant reductions, measuring 0.35 A_420_/g for the CH-group fruit salad and 0.27 A_420_/g for the CHBE-group fruit salad. CH and CHBE groups exhibited minimized moisture loss and color alteration in individual fruit, leading to diminished browning indices.

These edible coatings effectively curbed browning in fruit salads; the CH coating achieved this by mitigating fruit exposure to oxygen and moisture, slowing oxidation and enzymatic browning reactions, bolstered by chitosan’s antimicrobial and antioxidant properties, which, in tandem with the coating’s moisture reduction effect, inhibited oxidative reactions. In contrast, the CHBE coating surpassed the CH group in reducing the browning index, with its bergamot juice powder extract’s phenolic compounds acting as potent antioxidants and enzyme inhibitors that countered oxidation and enzymatic browning more effectively, resulting in a more pronounced reduction in the browning index. This study underscores the efficiency of CH and CHBE coatings in managing fruit color changes and reducing browning indices, with the added value of bergamot juice powder extract’s antioxidative properties enhancing the CHBE coating’s performance. This aligns with prior research highlighting the efficacy of edible coatings in mitigating browning and enhancing visual appeal in fresh-cut fruit through various compounds [34,35,36], including natural browning inhibitors like bergamot juice powder, thus contributing to the overall quality and shelf-life extension of fresh-cut fruit.

### 3.6. Polyphenol Oxidase Enzyme Activity

Enzymatic browning in fresh-cut produce, induced by mechanical processes like cutting, is mainly catalyzed by polyphenol oxidase (PPO) enzymes (EC 1.14.18.1), which oxidize phenolic substrates to *o*-quinones in the presence of oxygen. While PPO’s role in polyphenol oxidation is crucial for browning reactions, peroxidase (POD) also contributes to enzymatic browning on fresh-cut surfaces [1]. Furthermore, PPO has been associated with degrading phenols, vitamin C, flavonoids, and anthocyanins, resulting in nutrient loss [37].

During the initial storage day, significant variations in PPO enzyme activity were observed among the groups. The NC group exhibited lower activity (1.04 U mL^−1^) in fruit salads, while the CH-coated (3.47 U mL^−1^) and CHBE-coated (3.46 U mL^−1^) fruit salads displayed higher activity (Table 2). Without coatings, natural enzymatic browning processes were prominent in the NC group. Although initially less pronounced, the CH and CHBE coatings gradually limited oxygen and moisture permeability, thus slowing down oxidative reactions. This gradual impact is reflected in the increased PPO enzyme activity observed in the CH and CHBE groups on the first day. PPO enzyme activity in the NC group increased throughout storage due to natural fruit ripening, peaking at 19.48 U mL^−1^ by the storage endpoint. The CH coating effectively reduced PPO enzyme activity by 75.50% during storage, primarily by inhibiting oxidative reactions through reduced permeability to oxygen and moisture. In contrast, the CHBE coating, enriched with phenolic compounds displaying antioxidant and enzyme-inhibiting properties, more robustly suppressed oxidative reactions, resulting in a significant decline in PPO enzyme activity (83.53%).

Numerous studies have emphasized the ability of edible coatings to decrease PPO activity in fresh-cut fruit, such as strawberries and sapotas [38,39]. Coatings containing α-tocopherol effectively reduced PPO enzyme activity in apples [40]. Due to oxygen barrier effects, chitosan-coated strawberries exhibited lower PPO enzyme activity [41]. Reduced PPO enzyme activity corresponds to fewer enzymatic browning reactions, contributing to color preservation, freshness, and appearance. These effects align with individual fruit color values and the browning index of fruit salads. As a result, the influence of CH and CHBE coatings on PPO enzyme activity underscores their potential to uphold fruit quality and prolong shelf life, providing insights into enzymatic browning mitigation through edible coatings on fresh-cut produce.

### 3.7. Titratable Acidity and pH

Titratable acidity and pH values are crucial indicators of fresh-cut fruit’s acidic nature and acid–base equilibrium. Titratable acidity reveals the presence of free or bound acids in fruit, typically higher in acidic fruits. Conversely, pH signifies whether a fruit is acidic, basic, or neutral, often within the mildly acidic to neutral range.

During storage, all sample groups exhibited declining titratable acidity and increasing pH levels (Table 2). The NC group consistently had the highest titratable acidity values, while the lowest values were observed in the CHBE group. Similarly, the CHBE group displayed the highest pH values, contrasting with the lowest pH values found in the NC group. Without coating, organic acid breakdown accelerated due to increased respiration, leading to a 71.34% decrease in titratable acidity and a 17.08% increase in pH in the NC-group fruit salads. The CH coating’s barrier effects on oxygen and moisture slowed organic acid breakdown, resulting in a more gradual decrease in titratable acidity (26.00%) and a modest 2.45% pH increase. The CHBE coating, enriched with phenolic compounds, further stabilized the reduction in titratable acidity (10.14%) and limited the pH increase (from 3.67 to 3.73) compared to the CH group, possibly influenced by chitosan’s alkaline nature and bergamot juice powder’s acidic effect.

Edible coatings effectively maintained titratable acidity and pH values throughout storage, exhibiting a clear contrast to the NC group. These coatings regulated respiration rates and enzymatic reactions by mitigating gas exchange and moisture loss, sustaining acidity and pH equilibrium, a consistent trend according to prior research [3]. Similarly, the work of Muley and Singhal [42] underscored the efficacy of edible coatings in curbing pH and color alterations in strawberries, aiming for enhanced shelf life. The observed titratable acidity trends were in harmony with total soluble solids values and moisture preservation within fruit salads. As storage progressed, titratable acidity declined alongside an increase in soluble solid content, aligning with fruit maturation. This process led to reduced acidity and heightened sweetness, resulting in lower titratable acidity and elevated soluble solids—a pivotal aspect of fruit quality. Moreover, preserving optimal moisture levels contributed to stable juice concentration, thereby supporting titratable acidity and soluble solids—a vital combination for superior fruit taste and flavor. Striking a harmonious balance between acidity and sweetness is a critical determinant in shaping the overall sensory profile of fruit [29].

### 3.8. Microbial Quality

The natural physical and chemical barriers in a fruit’s epidermis, which inhibit microbial growth on the surface, become compromised during fresh-cut processing. This exposes fresh-cut fruit to an environment conducive to microbial growth due to their broad-cut surfaces serving as rich nutrient sources and elevated moisture and sugar levels [2]. Ensuring the microbiological safety of such products requires monitoring populations of mesophilic bacteria, psychotropic bacteria, molds, and yeasts throughout storage [29].

Edible coatings have emerged as a potential strategy to enhance product safety by acting as carriers for antimicrobial compounds that inhibit microbial growth on fresh-cut fruit [4]. These coatings create a protective barrier on the fruit surface, impeding the proliferation, dissemination, and colonization of microorganisms, thereby mitigating the risk of microbial spoilage [5].

**Figure 2:**
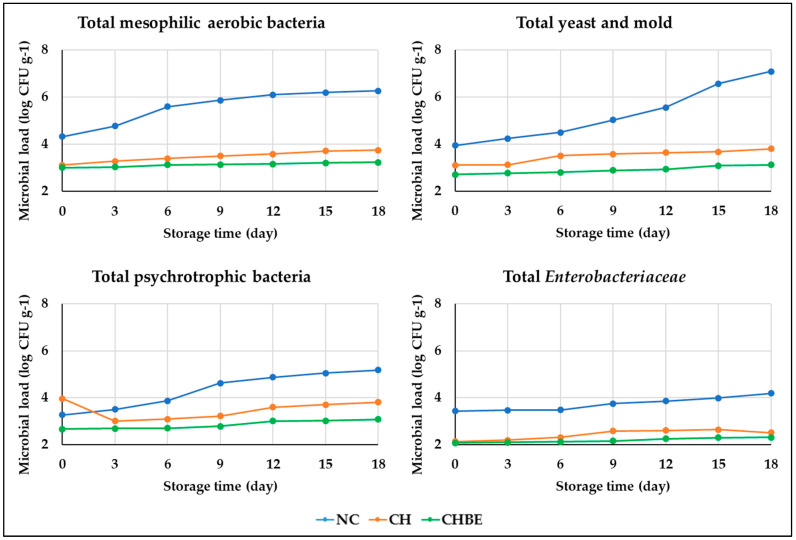
Microbial load (log CFU g^−1^) of fruit salads during storage (NC: non-coated; CH: chitosan-coated; CHBE: bergamot juice powder extract-enriched chitosan-coated).

In our storage study, TMAB, TPB, TYM, and TEN count changes were observed for all sample groups (Figure 2 and Table S3).

The NC group showed initial microbial loads of 4.33, 3.26, 3.94, and 3.43 log CFU g^−1^, increasing by 44.08%, 58.90%, 79.95%, and 22.16% by the end of storage. The CH group displayed restrained microbial growth, with post-storage increases of 20.51%, 28.72%, 22.58%, and 17.37% for TMAB, TPB, TYM, and TEN counts, attributed to reduced oxygen and moisture permeability. The CHBE coating, enriched with antimicrobial phenolic compounds from bergamot juice powder extract, exhibited superior microbial inhibition, consistently maintaining a lower microbial burden than the CH group. The CHBE group demonstrated increases of 7.64%, 15.41%, 15.19%, and 11.11% for TMAB, TPB, TYM, and TEN counts during storage, underscoring its remarkable microbial inhibition efficacy, corroborating findings by Pham et al. [3].

Researchers have effectively developed antimicrobial coatings by incorporating natural antimicrobial compounds into matrices based on proteins or polysaccharides [32]. Several studies have verified the efficacy of antimicrobial agents like organic acids (e.g., acetic, benzoic, lactic, propionic, sorbic), organic acid salts (e.g., potassium sorbate, sodium benzoate), fatty acid esters (such as glyceryl monolaurate), polypeptides (including lysozyme, peroxidase, lactoferrin, nisin), volatile and essential oils, spice extracts, nitrites, and sulfites in managing the growth of spoilage and pathogenic microorganisms in fresh-cut products [1].

The effectiveness of edible coatings in preserving the microbial quality of fresh-cut fruit is well documented. For instance, papayas coated with chitosan suppressed bacterial counts, mold, and yeast growth [17]. In apples, adding essential oils in edible coatings ultimately hindered natural microflora over a 14-day storage period [43]. Applying grape seed extract-enriched coatings on strawberries resulted in the lowest microbial growth during storage [44]. Coatings containing eugenol and citral reduced microbial growth in fresh-cut apples [45]. Alginate coatings enriched with antimicrobial compounds notably decreased microbial counts in fresh-cut pineapple [46]. Kiwis coated with chitosan-based solutions demonstrated reduced microbial growth for up to 12 days [47].

Although regulations for microbial loads exist for fruits and vegetables in the United States and Europe, specific guidelines for fresh-cut products are still in development. Typically, the aerobic microbial count should not surpass 10^6^ CFU g^−1^ by the product’s expiration date [5]. In our study, only the NC group exceeded this threshold from the ninth day onwards, while the coated groups consistently maintained TMAB counts well below the limit throughout storage.

Changes in microbial loads during fruit salad storage are closely linked to fruit characteristics like titratable acidity, pH, moisture, and total soluble solids content. The NC group’s high moisture and total soluble solids values create a favorable environment for microbial proliferation due to increased water activity and sugar content. In contrast, coatings like CH and CHBE with lower moisture content exhibit protective effects against microbial growth, leveraging their antimicrobial properties. Moreover, lower total soluble solids values indicate reduced microbial nutrition. Overall, these findings underscore the positive influence of coatings on microbial loads in conjunction with moisture, total soluble solid content, titratable acidity, and pH values. This underscores the efficacy of CH and CHBE coatings in preserving fruit quality by effectively managing microbial growth, thus extending freshness during storage.

### 3.9. Weight Loss

Weight loss during the storage of fresh-cut fruit directly impacts fruit quality and shelf life by reflecting natural ripening and deterioration processes, serving as an indicator of freshness, primarily driven by water loss. In our study, weight loss increased during storage across all sample groups (Table 2), consistent with observations by Guerreiro et al. [45] in fresh-cut apples.

The NC group of fruit salads experienced continuous weight loss throughout storage, ranging from 1.75% to 6.61%, indicating significant water loss and heightened susceptibility to deterioration. This aligns with the common weight loss phenomenon during fresh-cut fruit’s shelf life, mainly attributed to water migration [48]. Conversely, coated groups displayed reduced and stable weight loss rates. The CH group exhibited limited weight loss (2.45%), indicating the protective role of the coating in preserving surface moisture and freshness, supported by Jongsri et al. [25]. The most effective outcome was observed in the CHBE group, showcasing the lowest weight loss (2.10%). The antimicrobial properties of bergamot juice powder extract contributed to microbial control and reduced water loss, maintaining freshness. While the CH coating curbed water loss, the CHBE coating excelled by addressing microbial growth and weight loss. These findings underscore the potential of edible coatings in enhancing fruit salad quality and extending shelf life. Chong et al. [49] have demonstrated that chitosan coatings effectively minimize weight loss in stored fresh-cut fruit like apples, pears, strawberries, cantaloupes, and papayas. These coatings act as moisture barriers, addressing the critical cause of weight loss. Others have reached similar conclusions [2,5]. Monitoring coated fruit weight changes during storage is vital in assessing the moisture retention efficacy of coatings, as weight loss primarily reflects water loss.

### 3.10. Sensory Evaluation

**Figure 3:**
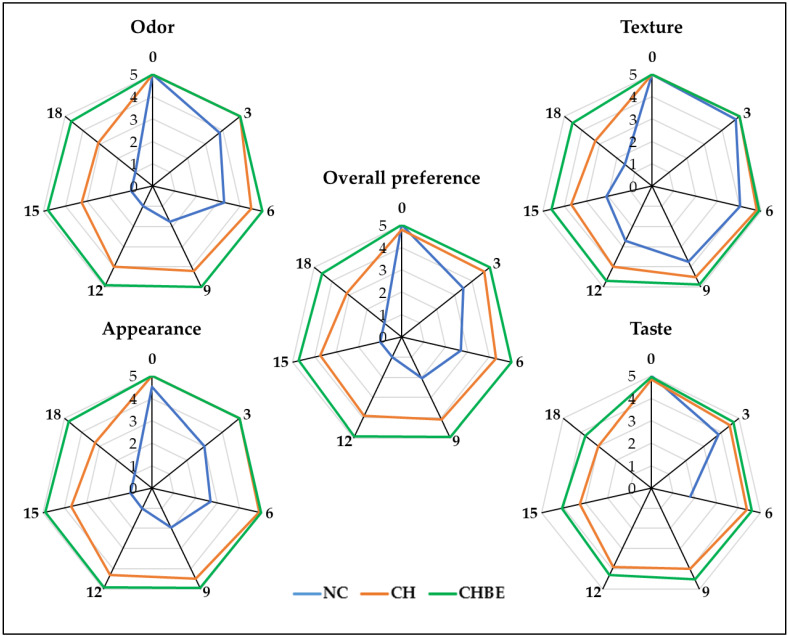
Sensory scores (rating out of 5) of fruit salads during storage (NC: non-coated; CH: chitosan-coated; CHBE: bergamot juice powder extract-enriched chitosan-coated).

Sensory attributes, including odor, texture, appearance, taste, and overall preference, play a crucial role in evaluating the quality of fresh-cut fruit and determining consumer satisfaction. Changes in sensory characteristics during storage are influenced by physiological and chemical shifts such as ripening, water loss, color changes, and microbial activities. The sensory evaluation scores of the samples on the 0, 3rd, 6th, 9th, 12th, 15th, and 18th day of storage are given in Figure 3 and Table S4.

In our study, the NC group exhibited the lowest scores attributed to the absence of protective coatings on fresh-cut fruit. This led to surface deterioration and noticeable sensory decline, particularly in taste. This trend is consistent with prior research emphasizing the quicker degradation of flavor compared to appearance. In contrast, the CH and CHBE groups consistently maintained higher scores, reflecting positive consumer evaluations during storage, a trend observed in other studies [2]. Ozdemir and Gokmen [50] reported sustained sensory quality of ascorbic acid-enriched chitosan-coated pomegranate arils even after 25 days at 5 °C. Motamedi et al. [51] demonstrated improved acceptability of coated oranges stored for 56 days at 4 °C, particularly regarding color, juiciness, odor, and taste.

In our study’s CHBE group, using bergamot juice powder extract did not compromise perception; it maintained high scores. Consumers detected mild acidity without the typical odor of bergamot, showcasing successful bioactive utilization without taste or aroma interference. The correlation between physicochemical analyses and sensory scores highlighted the effective management of sensory changes during storage with edible coatings. This preservation extended to odor, texture, appearance, taste, and overall preference, enhancing consumer satisfaction. The consistent superiority of the CHBE group underscored the significant impact of edible coatings in maintaining fruit salad freshness, emphasizing their value in ensuring quality and consumer contentment in the long-term storage of fruits and vegetables in the food industry.

## 4. Discussion

In summarizing the generalized outcomes of our study, several key findings emerge, emphasizing the efficacy of chitosan coatings enriched with bergamot juice powder in enhancing the quality and extending the shelf life of fresh-cut fruit salads. The comprehensive analyses conducted have provided valuable insights into various parameters, highlighting the multifunctional nature of this innovative coating approach. These findings are detailed below:

- Decay percentage and shelf life: The observed decay percentage provides valuable insights into the effectiveness of the chitosan coating enriched with bergamot juice powder in extending the shelf life of fresh-cut fruit salads. The lower decay percentage in the coated groups, particularly the CHBE group, highlights the preservative capabilities of the coating. This aligns with previous studies that have demonstrated the ability of chitosan-based coatings to inhibit microbial growth and delay decay in various fruits [17,38,39];
- Moisture content and weight loss: Moisture content and weight loss are crucial parameters affecting the overall quality of fresh-cut fruit. The reduced weight loss in the CHBE-coated group suggests that the bergamot juice powder extract contributes to microbial control but also aids in moisture retention. This dual functionality emphasizes the multifaceted benefits of incorporating natural extracts into edible coatings;
- Total soluble solids content, titratable acidity, and pH: Changes in total soluble solids content, titratable acidity, and pH indicate the impact of coatings on fruit maturation and flavor. The gradual increase in total soluble solids and the moderate reduction in titratable acidity in coated groups suggest a controlled and balanced maturation process. The preservation of optimal moisture levels, pH regulation, and the antimicrobial properties of the coatings likely contribute to maintaining desirable flavor profiles;
- Color and browning index: The significant reduction in the browning index and the preservation of color attributes in coated groups underscore the efficacy of chitosan coatings enriched with bergamot juice powder in mitigating enzymatic browning. This aligns with studies emphasizing the role of edible coatings in controlling color changes in various fruits [28,33];
- Polyphenol oxidase enzyme activity: The decline in polyphenol oxidase enzyme activity in the coated groups reflects the effectiveness of the coatings in inhibiting enzymatic browning reactions. This outcome is consistent with previous research showcasing the ability of chitosan-based coatings to reduce PPO activity and extend the shelf life of fresh-cut produce [40,41];
- Microbial quality: The antimicrobial properties of bergamot juice powder extract in the CHBE-coated group contributed to superior microbial inhibition throughout storage. This aligns with a growing body of evidence supporting using natural antimicrobial compounds in edible coatings to enhance the microbiological safety of fresh-cut products [1,5,32];
- Sensory evaluation: The sustained high scores in sensory evaluation, particularly in taste and overall preference, highlight the positive impact of chitosan coatings enriched with bergamot juice powder on consumer satisfaction. The absence of undesirable taste or aroma interference from bergamot’s typical odor further accentuates the bioactive potential of such coatings.

These findings collectively suggest that chitosan coatings enriched with bergamot juice powder can serve as a promising strategy for enhancing the quality and extending the shelf life of fresh-cut fruit salads. Gautam et al. [52] emphasized the significant potential of controlled release of bioactive compounds in edible films and coatings in enhancing food products’ shelf life and safety. The multifunctional nature of these coatings, addressing decay, microbial growth, enzymatic browning, and sensory attributes, positions them as valuable tools in the food industry for preserving the freshness and desirability of fruits. Furthermore, incorporating bergamot juice powder extract introduces a novel dimension, hinting at its potential as a natural and bioactive component in edible coatings, with the added benefit of pH regulation.

## 5. Conclusions

This study comprehensively evaluated the effects of edible coatings on the quality parameters of fresh-cut fruit salads during storage, with a specific focus on utilizing bergamot juice, an often overlooked byproduct. Despite being considered waste, bergamot juice possesses a rich bioactive profile. The innovation introduced in this study involves the conversion of bergamot juice into a dried powder form, revealing its potential as a protective component in edible coatings. Adding bergamot juice powder extract to chitosan coatings demonstrated substantial improvements. Notably, it effectively reduced decay percentages, showcasing its promising role in preserving the freshness of fruit salads. Additionally, this coating formulation contributed to elevated moisture content, soluble solids, and enhanced color attributes, ultimately improving visual appeal and taste. The coatings effectively prevented enzymatic browning, as evidenced by lower browning indices and reduced polyphenol oxidase activity. Interestingly, the enhanced microbiological safety observed in the bergamot juice powder extract-enriched chitosan-coated group emphasizes the multifunctional benefits of bergamot juice powder extract. Positive sensory evaluations further confirmed the overall superiority of this innovative approach. By repurposing bergamot juice waste as a dried powder, this study highlights a sustainable solution and introduces a valuable resource for post-harvest preservation practices within the food industry. The rich bioactive content of bergamot juice, previously deemed waste, has now emerged as a potential asset in enhancing fresh-cut fruit salads’ quality and shelf life through tailored edible coatings.

## Figures and Tables

**Table 1:** Decay percentage, moisture, and total soluble solids of fruit salads during storage (NC: non-coated; CH: chitosan-coated; CHBE: bergamot juice powder extract-enriched chitosan-coated) ^1^.

	Fresh-Cut Fruit	Sample Group	Storage Time (day)						
			0	3	6	9	12	15	18
Decay percentage (%)	Apple	NC	0 ± 0.00	73.33 ± 10.00 ^a^	86.67 ± 6.67 ^a^	93.33 ± 3.33 ^a^	100.00 ± 0.00 ^a^	100.00 ± 0.00 ^a^	100.00 ± 0.00 ^a^
		CH	0 ± 0.00	6.67 ± 3.33 ^d^	20.00 ± 8.67 ^d^	33.33 ± 6.67 ^d^	40.00 ± 3.33 ^e^	46.67 ± 3.33 ^d^	53.33 ± 6.67 ^b^
		CHBE	0 ± 0.00	0 ± 0.00 ^e^	0 ± 0.00 ^e^	6.67 ± 3.33 ^e^	20.00 ± 6.67 ^h^	26.67 ± 3.33 ^g^	33.33 ± 3.33 ^e^
	Pear	NC	0 ± 0.00	26.67 ± 3.33 ^b^	73.33 ± 10.00 ^b^	86.67 ± 5.77 ^a^	93.33 ± 4.71 ^b^	100.00 ± 0.00 ^a^	100.00 ± 0.00 ^a^
		CH	0 ± 0.00	6.67 ± 0.10 ^d^	13.33 ± 2.00 ^d^	26.67 ± 1.50 ^d^	33.33 ± 3.00 ^f^	40.00 ± 1.00 ^e^	46.67 ± 1.75 ^c^
		CHBE	0 ± 0.00	0 ± 0.00 ^e^	0 ± 0.00 ^e^	6.67 ± 0.33	13.33 ± 4.71 ^h^	20.00 ± 3.06 ^h^	26.67 ± 3.06 ^ef^
	Pineapple	NC	0 ± 0.00	0 ± 0.00 ^e^	40 ± 4.00 ^c^	46.67 ± 9.08 ^c^	60.00 ± 5.56 ^d^	73.33 ± 4.84 ^c^	100.00 ± 0.00 ^a^
		CH	0 ± 0.00	0 ± 0.00 ^e^	0 ± 0.00 ^e^	0 ± 0.00 ^e^	20.00 ± 3.33 ^h^	26.67 ± 0.00 ^g^	26.67 ± 0.00 ^ef^
		CHBE	0 ± 0.00	0 ± 0.00 ^e^	0 ± 0.00 ^e^	0 ± 0.00 ^e^	6.67 ± 3.06 ^i^	13.33 ± 1.18 ^i^	13.33 ± 2.00 ^g^
	Kiwi	NC	0 ± 0.00	13.33 ± 3.33 ^c^	46.67 ± 0.13 ^c^	73.33 ± 3.10 ^b^	80.00 ± 1.50 ^c^	86.67 ± 1.00 ^b^	100.00 ± 0.00 ^a^
		CH	0 ± 0.00	0 ± 0.00 ^e^	0 ± 0.00 ^e^	6.67 ± 0.00 ^e^	26.67 ± 0.33 ^g^	33.33 ± 1.00 ^f^	40.00 ± 10.16 ^d^
		CHBE	0 ± 0.00	0 ± 0.00 ^e^	0 ± 0.00 ^e^	6.67 ± 0.00 ^e^	13.33 ± 0.67 ^h^	13.33 ± 4.17 ^i^	20.00 ± 0.50 ^f^
Moisture content (%)	Apple	NC	84.58 ± 1.20 ^f^	82.56 ± 0.90 ^e^	81.25 ± 0.20 ^f^	80.53 ± 0.74 i	80.10 ± 0.84 ^g^	78.70 ± 0.10 ^f^	75.50 ± 1.05 ^g^
		CH	88.75 ± 0.55 ^c^	87.67 ± 0.70 ^c^	87.20 ± 0.50 ^d^	87.13 ± 1.05 ^e^	87.12 ± 0.94 ^d^	87.11 ± 1.06 ^d^	86.15 ± 0.41 ^d^
		CHBE	89.18 ± 0.85 ^bc^	88.69 ± 0.48 ^bc^	88.12 ± 0.68 ^cd^	87.69 ± 0.17 ^de^	87.50 ± 0.54 ^d^	87.12 ± 1.05 ^d^	87.05 ± 1.52 ^cd^
	Pear	NC	86.72 ± 0.70 ^e^	84.39 ± 0.66 ^d^	83.10 ± 0.58 ^e^	82.52 ± 0.34 ^g^	81.06 ± 0.90 ^fg^	80.72 ± 0.78 ^e^	78.19 ± 0.80 ^f^
		CH	90.02 ± 0.35 ^ab^	89.60 ± 0.20 ^ab^	89.12 ± 1.05 ^bc^	88.59 ± 1.81 ^bcd^	88.10 ± 0.99 ^cd^	87.92 ± 0.76 ^cd^	87.81 ± 0.30 ^bc^
		CHBE	90.05 ± 0.64 ^ab^	89.71 ± 0.48 ^ab^	89.52 ± 0.27 ^ab^	88.35 ± 0.11 ^cde^	88.29 ± 0.19 ^bcd^	88.17 ± 0.20 ^cd^	88.02 ± 0.58 ^bc^
	Pineapple	NC	88.00 ± 0.41 ^cd^	87.62 ± 0.52 ^c^	86.63 ± 0.87 ^d^	84.58 ± 0.17 ^f^	83.06 ± 0.69 ^e^	81.92 ± 0.58 ^e^	80.63 ± 0.44 ^e^
		CH	90.93 ± 1.05 ^a^	90.78 ± 0.57 ^a^	90.12 ± 1.20 ^ab^	89.93 ± 0.65 ^ab^	89.59 ± 0.58 ^ab^	89.48 ± 0.48 ^ab^	89.10 ± 0.72 ^ab^
		CHBE	91.26 ± 0.29 ^a^	90.82 ± 0.03 ^a^	90.54 ± 0.84 ^a^	90.17 ± 0.74 ^a^	90.08 ± 1.03 ^a^	89.99 ± 1.00 ^a^	89.64 ± 0.23 ^a^
	Kiwi	NC	87.10 ± 0.58 ^de^	84.99 ± 0.47 ^d^	83.69 ± 0.10 ^e^	83.10 ± 0.36 ^g^	81.69 ± 0.90 ^f^	81.06 ± 0.45 ^e^	79.29 ± 0.25 ^f^
		CH	90.45 ± 0.59 ^a^	90.12 ± 1.05 ^a^	90.02 ± 0.80 ^ab^	89.64 ± 0.66 ^abc^	89.10 ± 0.67 ^abc^	88.73 ± 0.31 ^bc^	88.34 ± 0.74 ^abc^
		CHBE	90.49 ± 0.57 ^a^	90.28 ± 0.95 ^a^	90.21 ± 0.58 ^ab^	89.91 ± 0.10 ^ab^	89.12 ± 0.18 ^abc^	89.02 ± 0.52 ^abc^	88.67 ± 0.70 ^ab^
Total soluble solid content (%)	Apple	NC	15.42 ± 0.01 ^a^	14.28 ± 0.03 ^a^	13.58 ± 0.05 ^a^	15.43 ± 0.01 ^b^	15.48 ± 0.01 ^c^	15.79 ± 0.02 ^b^	15.82 ± 0.03 ^b^
		CH	12.54 ± 0.12 ^e^	12.57 ± 0.04 ^e^	13.15 ± 0.10 ^bc^	13.57 ± 0.08 ^d^	13.84 ± 0.07 ^e^	13.99 ± 0.03 ^c^	14.03 ± 0.01 ^c^
		CHBE	11.87 ± 0.06 ^f^	12.03 ± 0.18 ^f^	12.39 ± 0.10 ^e^	12.81 ± 0.07 ^e^	12.94 ± 0.05 ^g^	13.02 ± 0.13 ^e^	13.26 ± 0.25 ^de^
	Pear	NC	14.55 ± 0.09 ^b^	13.83 ± 0.10 ^b^	13.29 ± 0.06 ^b^	15.62 ± 0.14 ^b^	15.73 ± 0.05 ^b^	15.89 ± 0.02 ^b^	16.01 ± 0.02 ^b^
		CH	11.95 ± 0.01 ^f^	12.05 ± 0.01 ^f^	12.09 ± 0.01 ^f^	12.24 ± 0.06 ^f^	13.25 ± 0.24 ^f^	13.36 ± 0.08 ^d^	13.39 ± 0.17 ^d^
		CHBE	11.25 ± 0.06 ^g^	11.49 ± 0.05 ^g^	11.54 ± 0.27 ^h^	11.89 ± 0.11 ^g^	12.45 ± 0.18 ^h^	12.60 ± 0.05 ^f^	13.05 ± 0.02 ^e^
	Pineapple	NC	14.02 ± 0.01 ^d^	13.24 ± 0.06 ^d^	12.70 ± 0.07 ^d^	14.89 ± 0.37 ^c^	15.05 ± 0.18 ^d^	16.17 ± 0.20 ^a^	17.18 ± 0.14 ^a^
		CH	10.31 ± 0.05 ^h^	10.86 ± 0.08 ^i^	11.52 ± 0.17 ^h^	11.57 ± 0.19 ^h^	12.10 ± 0.20 ^i^	12.61 ± 0.08 ^f^	12.53 ± 0.03 ^fg^
		CHBE	10.17 ± 0.01 ^i^	10.81 ± 0.05 ^i^	11.08 ± 0.06 ^i^	11.16 ± 0.04 ^i^	11.87 ± 0.14 ^j^	12.05 ± 0.14 ^g^	12.40 ± 0.05 ^g^
	Kiwi	NC	14.36 ± 0.03 ^c^	13.62 ± 0.10 ^c^	13.03 ± 0.15 ^c^	16.04 ± 0.23 ^a^	16.09 ± 0.04 ^a^	16.13 ± 0.07 ^a^	17.05 ± 0.31 ^a^
		CH	10.36 ± 0.06 ^h^	11.42 ± 0.09 ^g^	11.87 ± 0.16 ^g^	12.01 ± 0.10 ^fg^	12.35 ± 0.02 ^h^	12.72 ± 0.03 ^f^	12.68 ± 0.05 ^f^
		CHBE	10.28 ± 0.05 ^h^	11.03 ± 0.17 ^h^	11.29 ± 0.10 ^i^	11.45 ± 0.11 ^h^	12.01 ± 0.05 ^ij^	12.54 ± 0.22 ^f^	12.57 ± 0.04 ^fg^

^1^ Different letters in the same column (based on each analysis) indicate significant differences among the sample groups according to Duncan’s test (*p* < 0.05).

**Table 2:** Browning index, polyphenol oxidase enzyme activity, titratable acidity, pH, and weight loss values of fruit salads during storage (NC: non-coated; CH: chitosan-coated; CHBE: bergamot juice powder extract-enriched chitosan-coated) ^1^.

Analyte	Sample Group	Storage Time (day)						
		0	3	6	9	12	15	18
Browning index (A_420_/g)	NC	0.38 ± 0.02 ^a^	0.38 ± 0.03 ^a^	0.40 ± 0.05 ^a^	0.42 ± 0.01 ^a^	0.45 ± 0.06 ^a^	0.54 ± 0.02 ^a^	0.70 ± 0.05 ^a^
	CH	0.22 ± 0.01 ^b^	0.25 ± 0.02 ^b^	0.28 ± 0.02 ^b^	0.30 ± 0.05 ^b^	0.31 ± 0.07 ^b^	0.35 ± 0.08 ^b^	0.35 ± 0.01 ^b^
	CHBE	0.19 ± 0.03 ^b^	0.20 ± 0.01 ^c^	0.23 ± 0.01 ^ab^	0.27 ± 0.01 ^b^	0.28 ± 0.02 ^b^	0.29 ± 0.04 ^b^	0.27 ± 0.01 ^c^
Polyphenol oxidase enzyme activity (U mL^−1^)	NC	1.04 ± 0.10 ^b^	4.93 ± 0.13 ^a^	5.41 ± 0.04 ^a^	6.93 ± 0.15 ^a^	10.57 ± 0.10 ^a^	13.87 ± 0.05 ^a^	19.48 ± 0.04 ^a^
	CH	3.47 ± 0.04 ^a^	2.56 ± 0.10 ^b^	1.89 ± 0.16 ^b^	1.77 ± 0.10 ^b^	1.03 ± 0.05 ^b^	0.94 ± 0.07 ^b^	0.85 ± 0.03 ^b^
	CHBE	3.46 ± 0.12 ^a^	2.12 ± 0.11 ^c^	1.07 ± 0.04 ^c^	1.00 ± 0.01 ^c^	0.82 ± 0.01 ^c^	0.69 ± 0.13 ^c^	0.57 ± 0.08 ^c^
Titratable acidity (%)	NC	1.57 ± 0.02 ^a^	1.33 ± 0.30 ^b^	1.30 ± 0.10 ^a^	1.27 ± 0.04 ^b^	0.87 ± 0.07 ^b^	0.50 ± 0.09 ^c^	0.45 ± 0.10 ^c^
	CH	1.50 ± 0.05 ^a^	1.47 ± 0.02 ^a^	1.46 ± 0.11 ^a^	1.45 ± 0.10 ^a^	1.43 ± 0.03 ^a^	1.20 ± 0.04 ^b^	1.11 ± 0.05 ^b^
	CHBE	1.48 ± 0.20 ^a^	1.50 ± 0.10 ^a^	1.46 ± 0.07 ^a^	1.40 ± 0.06 ^ab^	1.37 ± 0.13 ^a^	1.35 ± 0.04 ^a^	1.33 ± 0.07 ^a^
pH	NC	3.63 ± 0.10 ^a^	3.65 ± 0.11 ^a^	3.66 ± 0.08 ^a^	3.85 ± 0.09 ^a^	3.86 ± 0.75 ^a^	3.95 ± 0.70 ^a^	4.25 ± 0.06 ^a^
	CH	3.68 ± 0.10 ^a^	3.70 ± 0.08 ^a^	3.71 ± 0.05 ^a^	3.71 ± 0.33 ^a^	3.74 ± 0.42 ^a^	3.76 ± 0.61 ^a^	3.77 ± 0.50 ^a^
	CHBE	3.67 ± 0.15 ^a^	3.68 ± 0.50 ^a^	3.69 ± 0.40 ^a^	3.69 ± 0.22 ^a^	3.71 ± 0.11 ^a^	3.72 ± 0.07 ^a^	3.73 ± 0.05 ^a^
Weight loss (%)	NC	0 ± 0.00	1.75 ± 0.01 ^a^	3.85 ± 0.03 ^a^	4.64 ± 0.05 ^a^	5.10 ± 0.05 ^a^	6.43 ± 0.04 ^a^	6.61 ± 0.06 ^a^
	CH	0 ± 0.00	0.36 ± 0.02 ^b^	0.58 ± 0.01 ^b^	0.96 ± 0.05 ^b^	1.74 ± 0.01 ^b^	2.30 ± 0.08 ^b^	2.45 ± 0.07 ^b^
	CHBE	0 ± 0.00	0.31 ± 0.01 ^c^	0.43 ± 0.02 ^c^	0.80 ± 0.03 ^c^	1.54 ± 0.04 ^c^	1.78 ± 0.03 ^c^	2.10 ± 0.02 ^c^

^1^ Different letters in the same column (based on each analysis) indicate significant differences among the sample groups according to Duncan’s test (*p* < 0.05).

## Data Availability

Data will be available upon reasonable request.

## Supplementary Materials

The following supporting information can be downloaded at: https://www.mdpi.com/article/10.3390/foods13010147/s1, Table S1: *L**, *a**, and *b** color coordinates of fruit salads during storage; Table S2. *WI**, Δ*E**, Δ*C**, and *CI** values of fruit salads during storage; Table S3. Microbial load (log CFU g^−1^) of fruit salads during storage; Table S4. Sensory scores (rating out of 5) of fruit salads during storage.
