# Supplementary material for: Control of Browning, Enzyme Activity, and Quality in Stored Fresh-cut Fruit Salads through Chitosan Coating Enriched with Bergamot Juice Powder
Source: Foods. 2024 Jan 1;13(1):147. doi: 10.3390/foods13010147 (PMC10779034; doi:10.3390/foods13010147)
Supplement: Supplementary file 1 [file foods-13-00147-s001.zip › foods-2801067-supplementary.pdf]

Table S1: *L**, *a**, and *b** color coordinates of fruit salads during storage (NC: non-coated; CH: chitosan-coated; CHBE: bergamot juice powder extract-enriched chitosan-coated)[1].

	Fresh-cut fruit	Sample group	Storage time (day)						
			**0**	**3**	**6**	**9**	**12**	**15**	**18**
L*	Apple	NC	79.07±0.36^{ab}	76.11±0.81^{abcd}	74.83±1.05^{abc}	70.74±2.04^{def}	68.26±0.10^{c}	62.26±0.57^{d}	62.01±0.68^{d}
		CH	80.23±2.05^{a}	78.10±1.47^{ab}	76.25±1.08^{ab}	75.10±1.80^{ab}	73.26±3.25^{ab}	71.59±0.24^{ab}	71.02±3.07^{ab}
		CHBE	80.30±1.58^{a}	78.29±2.54^{a}	77.26±0.74^{a}	76.26±0.69^{a}	75.05±0.18^{a}	73.10±0.38^{a}	72.10±1.74^{a}
	Pear	NC	75.39±0.54^{c}	73.99±0.71^{d}	71.75±0.06^{d}	69.53±0.20^{f}	65.05±0.85^{d}	62.99±1.05^{d}	59.41±1.64^{e}
		CH	76.65±2.28^{bc}	74.41±2.68^{cd}	73.26±3.48^{cd}	72.05±1.20^{cde}	71.05±1.47^{b}	70.52±0.58^{b}	68.52±0.21^{b}
		CHBE	77.15±0.69^{bc}	75.04±1.50^{cd}	74.05±1.47^{bcd}	72.98±2.05^{bcd}	71.82±2.04^{b}	71.07±0.25^{b}	69.41±0.06^{ab}
	Pineapple	NC	77.58±1.25^{bc}	77.10±2.25^{abc}	76.40±1.47^{ab}	70.15±2.01^{ef}	68.51±0.36^{c}	66.10±0.58^{c}	65.25±0.98^{c}
		CH	77.17±0.05^{bc}	78.89±0.85^{a}	76.05±1.05^{ab}	74.05±1.14^{abc}	71.05±1.65^{b}	70.20±1.00^{b}	69.57±0.25^{ab}
		CHBE	77.75±0.05^{bc}	75.38±0.07^{bcd}	74.14±0.25^{bcd}	73.84±0.64^{bc}	72.08±0.80^{b}	71.89±0.10^{ab}	71.05±0.58^{ab}
	Kiwi	NC	59.19±2.05^{d}	58.24±1.04^{e}	56.20±0.25^{e}	54.03±0.17^{h}	53.05±0.58^{f}	49.51±0.69^{f}	48.26±0.17^{g}
		CH	60.70±2.05^{d}	57.52±1.06^{e}	56.74±1.11^{e}	55.74±0.57^{gh}	55.01±0.47^{ef}	54.79±2.81^{e}	54.63±3.04^{f}
		CHBE	61.25±0.10^{d}	59.58±0.36^{e}	58.41±0.97^{e}	57.10±0.58^{g}	56.02±1.05^{e}	55.41±0.47^{e}	55.34±0.44^{f}
a*	Apple	NC	-6.85±0.05^{b}	-6.47±0.20^{b}	-6.10±0.30^{cd}	-5.25±0.14^{b}	-5.01±0.25^{d}	-4.14±0.41^{c}	-4.06±0.14^{d}
		CH	-7.45±0.20^{bc}	-7.14±0.35^{bc}	-7.01±0.64^{cde}	-6.25±0.17^{bc}	-5.78±0.18^{e}	-5.23±0.36^{cd}	-5.01±0.90^{e}
		CHBE	-9.01±0.05^{d}	-8.50±1.02^{cd}	-7.55±0.03^{de}	-6.66±1.05^{c}	-6.41±0.25^{f}	-6.34±1.7^{def}	-6.24±0.45^{f}
	Pear	NC	-1.49±1.25^{a}	0.12±1.03^{a}	0.42±0.58^{ab}	0.50±1.78^{a}	1.26±0.26^{b}	2.25±0.91^{ab}	5.45±0.25^{a}
		CH	-1.58±0.05^{a}	-1.19±1.03^{a}	-0.96±1.65^{b}	0.93±0.58^{a}	1.02±0.41^{b}	1.14±0.22^{b}	3.25±0.10^{b}
		CHBE	-1.73±1.20^{a}	-0.60±0.25^{a}	1.69±0.58^{a}	1.75±0.74^{a}	2.12±0.65^{a}	2.82±0.06^{a}	3.10±0.05^{b}
	Pineapple	NC	-7.06±0.02^{b}	-6.68±1.04^{b}	-5.83±0.58^{c}	-5.06±0.21^{b}	-4.26±0.14^{c}	-3.99±0.31^{c}	-2.58±0.33^{c}
		CH	-8.58±1.04^{cd}	-8.17±1.08^{cd}	-7.84±0.58^{e}	-6.80±0.54^{c}	-6.54±0.20^{f}	-6.12±0.10^{de}	-5.78±1.02^{ef}
		CHBE	-8.76±1.03^{cd}	-8.72±1.41^{d}	-8.18±1.80^{e}	-7.55±0.25^{cd}	-7.02±0.60^{f}	-6.95±0.80^{ef}	-6.18±0.15^{f}
	Kiwi	NC	-12.73±1.00^{e}	-12.72±0.25^{e}	-12.08±0.30^{f}	-8.66±0.64^{d}	-9.25±0.17^{g}	-7.49±0.80^{f}	-6.17±0.91^{f}
		CH	-14.58±1.02^{f}	-13.58±0.22^{e}	-12.84±0.47^{f}	-10.59±0.81^{e}	-9.99±0.33^{h}	-9.47±0.64^{g}	-8.47±0.40^{g}
		CHBE	-15.25±0.03^{f}	-14.05±0.07^{e}	-13.48±0.91^{f}	-13.12±0.42^{f}	-11.05±0.25^{i}	-10.58±0.17^{g}	-11.27±0.11^{h}
b*	Apple	NC	28.19±0.05^{g}	28.96±0.10^{g}	30.12±0.06^{e}	30.47±0.07^{f}	30.52±0.20^{e}	33.52±0.14^{d}	35.25±0.14^{c}
		CH	31.54±0.02^{e}	31.99±0.03^{e}	32.10±0.04^{d}	32.37±0.07^{d}	33.56±0.10^{c}	34.10±0.25^{c}	35.84±0.01^{b}
		CHBE	31.98±0.05^{d}	32.56±0.02^{d}	34.52±0.04^{c}	35.10±0.10^{b}	35.22±0.11^{b}	36.00±0.47^{a}	36.14±0.02^{a}
	Pear	NC	7.93±0.03^{l}	8.87±0.08^{l}	9.48±0.07^{j}	10.12±0.40^{k}	10.89±0.61^{i}	12.95±0.30^{j}	13.50±0.22^{j}
		CH	8.38±0.16^{k}	9.12±0.17^{k}	10.71±0.10^{i}	10.79±0.13^{j}	11.02±0.10^{i}	11.29±0.04^{k}	13.58±0.05^{j}
		CHBE	9.01±0.01^{j}	9.58±0.02^{j}	11.02±0.05^{h}	12.02±0.01^{i}	12.51±0.01^{h}	12.78±0.04^{j}	13.80±0.20^{i}
	Pineapple	NC	37.34±0.12^{c}	36.61±0.14^{c}	35.13±0.18^{b}	34.01±0.17^{c}	30.25±0.11^{e}	27.78±0.05^{h}	24.52±0.03^{h}
		CH	41.23±0.02^{b}	41.05±0.01^{a}	39.29±0.08^{a}	38.95±0.09^{a}	37.52±0.10^{a}	35.15±0.16^{b}	28.10±0.17^{g}
		CHBE	42.51±0.03^{a}	37.13±0.02^{b}	39.35±0.10^{a}	34.26±0.02^{c}	32.25±0.01^{d}	30.28±0.01^{f}	30.05±0.01^{f}
	Kiwi	NC	25.40±0.03^{i}	25.91±0.20^{i}	26.65±0.11^{g}	27.58±0.14^{h}	16.58±0.07^{g}	15.76±0.02^{i}	29.89±0.05^{f}
		CH	27.10±0.05^{h}	27.16±0.14^{h}	27.41±0.20^{f}	28.01±0.10^{g}	28.54±0.11^{f}	29.51±0.21^{g}	31.25±0.03^{e}
		CHBE	29.10±0.07^{f}	29.84±0.04^{f}	30.15±0.05^{e}	31.26±0.10^{e}	32.00±0.23^{d}	32.18±0.34^{e}	32.05±0.06^{d}

[1] Different letters in the same column (based on each analysis) indicate significant differences among the sample groups according to the Duncan's test ($P<0.05$).

Table S2. *WI**, Δ*E**, Δ*C**, and *CI** values of fruit salads during storage (NC: non-coated; CH: chitosan-coated; CHBE: bergamot juice powder extract-enriched chitosan-coated)[1].

	Fresh-cut fruit	Sample group	Storage time (day)						
			0	3	6	9	12	15	18
*WI**	Apple	NC	64.23±0.18^{b}	61.90±0.46^{b}	60.28±0.67^{b}	57.43±1.37^{cd}	55.68±0.04^{c}	49.35±0.36^{f}	48.02±0.41^{d}
		CH	62.04±1.09^{c}	60.58±0.85^{b}	59.46±0.71^{bc}	58.69±1.05^{c}	56.70±1.95^{c}	55.31±0.00^{d}	53.64±2.01^{c}
		CHBE	61.37±0.88^{c}	59.95±1.57^{b}	57.98±0.37^{cd}	57.11±0.46^{cd}	56.36±0.05^{c}	54.61±0.09^{de}	53.92±1.10^{c}
	Pear	NC	74.10±0.54^{a}	72.52±0.64^{a}	70.20±0.03^{a}	67.89±0.05^{b}	63.37±0.62^{b}	60.73±0.84^{b}	56.88±1.44^{b}
		CH	75.14±2.09^{a}	72.81±2.50^{a}	71.18±3.24^{a}	70.03±1.05^{a}	69.01±1.32^{a}	68.41±0.52^{a}	65.56±0.16^{a}
		CHBE	75.38±0.72^{a}	73.26±1.40^{a}	71.76±1.29^{a}	70.38±1.82^{a}	69.10±1.81^{a}	68.25±0.21^{a}	66.30±0.03^{a}
	Pineapple	NC	55.88±0.54^{d}	56.30±1.22^{c}	57.28±0.74^{d}	54.47±1.21^{e}	56.13±0.19^{c}	55.99±0.44^{d}	57.39±0.80^{b}
		CH	52.10±0.19^{e}	53.12±0.56^{d}	53.32±0.57^{e}	52.71±0.63^{f}	52.16±0.95^{d}	53.51±0.53^{e}	58.18±0.21^{b}
		CHBE	51.23±0.18^{e}	54.60±0.29^{cd}	52.21±0.36^{e}	56.24±0.41^{d}	56.77±0.61^{c}	58.10±0.19^{c}	57.82±0.41^{b}
	Kiwi	NC	50.27±1.92^{ef}	49.24±0.82^{e}	47.33±0.22^{f}	45.70±0.17^{g}	49.36±0.54^{e}	46.58±0.76^{g}	39.93±0.21^{f}
		CH	50.09±1.88^{ef}	47.78±0.85^{e}	47.20±0.92^{f}	46.56±0.58^{g}	45.79±0.39^{f}	45.19±2.31^{h}	44.26±2.52^{e}
		CHBE	49.20±0.45^{f}	47.83±0.27^{e}	46.89±0.96^{f}	45.32±0.50^{g}	44.50±0.75^{f}	44.00±0.21^{h}	43.89±0.34^{e}
Δ*E**	Apple	NC	Ns	Ns	Ns	Ns	Ns	Ns	Ns
		CH	3.83±0.49^{cd}	3.72±0.27^{bc}	2.61±0.12^{c}	4.86±0.22^{b}	6.11±2.50^{de}	9.41±0.31^{d}	9.12±2.27^{ab}
		CHBE	4.63±0.33^{abc}	4.91±0.42^{a}	5.24±0.08^{a}	7.40±1.16^{a}	8.38±0.10^{c}	11.38±0.36^{c}	10.37±0.95^{a}
	Pear	NC	Ns	Ns	Ns	Ns	Ns	Ns	Ns
		CH	1.97±1.17^{e}	2.06±0.67^{d}	3.60±1.38^{bc}	2.90±0.64^{c}	6.02±0.60^{de}	7.82±0.30^{ef}	9.38±1.35^{ab}
		CHBE	2.08±0.12^{e}	1.58±0.83^{d}	3.15±1.01^{c}	4.42±0.98^{b}	7.05±1.05^{cd}	8.13±0.85^{e}	10.29±1.49^{a}
	Pineapple	NC	Ns	Ns	Ns	Ns	Ns	Ns	Ns
		CH	4.38±0.36^{bc}	5.12±0.61^{a}	4.65±0.06^{ab}	6.55±0.66^{a}	8.09±0.38^{c}	8.70±0.34^{de}	6.48±0.75^{b}
		CHBE	5.58±0.44^{a}	3.17±0.95^{c}	5.51±0.18^{a}	4.50±1.15^{b}	4.96±0.03^{e}	6.97±0.62^{f}	8.79±0.20^{ab}
	Kiwi	NC	Ns	Ns	Ns	Ns	Ns	Ns	Ns
		CH	2.93±0.00^{d}	1.68±0.04^{d}	1.38±0.31^{d}	2.63±0.13^{c}	12.14±0.02^{b}	14.94±0.94^{b}	6.94±2.79^{b}
		CHBE	5.23±0.37^{ab}	4.39±0.30^{ab}	4.44±0.13^{ab}	6.55±0.32^{a}	15.81±0.23^{a}	17.73±0.33^{a}	8.99±0.67^{ab}
Δ*C**	Apple	NC	Ns	Ns	Ns	Ns	Ns	Ns	Ns
		CH	3.40±0.06^{c}	3.10±0.10^{c}	2.19±0.16^{c}	2.15±0.02^{c}	3.14±0.08^{f}	1.24±0.09^{f}	1.16±0.68^{d}
		CHBE	4.36±0.00^{b}	4.17±0.47^{b}	4.64±0.06^{a}	4.89±0.24^{b}	4.90±0.09^{d}	3.46±0.58^{d}	2.35±0.33^{c}
	Pear	NC	Ns	Ns	Ns	Ns	Ns	Ns	Ns
		CH	1.01±0.49^{e}	1.33±0.02^{e}	1.96±0.72^{c}	1.18±0.61^{d}	0.47±0.25^{h}	2.03±0.58^{e}	2.21±0.14^{c}
		CHBE	1.11±0.01^{e}	1.14±0.44^{e}	1.99±0.01^{c}	2.34±0.86^{c}	1.90±0.33^{g}	0.84±0.50^{f}	2.37±0.19^{c}
	Pineapple	NC	Ns	Ns	Ns	Ns	Ns	Ns	Ns
		CH	4.24±0.46^{b}	4.68±0.14^{a}	4.62±0.09^{a}	5.24±0.18^{ab}	7.62±0.03^{c}	7.67±0.16^{c}	4.83±0.35^{b}
		CHBE	5.49±0.40^{a}	2.10±0.39^{d}	4.90±0.65^{a}	2.50±0.05^{c}	3.41±0.43^{e}	3.88±0.40^{d}	6.60±0.08^{a}
	Kiwi	NC	Ns	Ns	Ns	Ns	Ns	Ns	Ns
		CH	2.51±0.00^{d}	1.52±0.03^{e}	1.08±0.06^{d}	1.98±0.17^{c}	11.98±0.03^{b}	13.89±0.21^{b}	2.68±0.43^{c}
		CHBE	4.52±0.57^{b}	4.15±0.09^{b}	3.79±0.28^{b}	5.78±0.14^{a}	15.52±0.15^{a}	16.71±0.43^{a}	5.54±0.74^{b}
*CI**	Apple	NC	-3.07±0.04^{a}	-2.94±0.13^{c}	-2.71±0.18^{cd}	-2.44±0.14^{b}	-2.40±0.14^{c}	-1.98±0.22^{c}	-1.86±0.09^{cd}
		CH	-2.94±0.16^{a}	-2.86±0.20^{c}	-2.86±0.31^{d}	-2.57±0.14^{b}	-2.35±0.18^{c}	-2.14±0.17^{c}	-1.97±0.44^{cd}
		CHBE	-3.51±0.09^{a}	-3.33±0.51	-2.83±0.04^{d}	-2.49±0.42^{b}	-2.43±0.11^{c}	-2.41±0.69^{cd}	-2.39±0.23^{de}
	Pear	NC	-2.49±2.12^{a}	0.18±1.57^{a}	0.62±0.85^{b}	0.71±2.50^{a}	1.78±0.24^{b}	2.76±1.01^{a}	6.80±0.01^{a}
		CH	-2.46±0.20^{a}	-1.75±1.62^{bc}	-1.22±2.18^{c}	1.20±0.71^{a}	1.30±0.48^{b}	1.43±0.26^{b}	3.49±0.08^{b}
		CHBE	-2.49±1.75^{a}	-0.83±0.37^{ab}	2.07±0.66^{a}	1.99±0.79^{a}	2.36±0.65^{a}	3.10±0.04^{a}	3.24±0.00^{b}
	Pineapple	NC	-2.44±0.05^{a}	-2.37±0.45^{c}	-2.17±0.27^{cd}	-2.12±0.16^{b}	-2.06±0.08^{c}	-2.17±0.19^{d}	-1.61±0.23^{c}
		CH	-2.70±0.33^{a}	-2.52±0.36^{c}	-2.62±0.24^{cd}	-2.36±0.23^{b}	-2.45±0.14^{c}	-2.48±0.09^{cd}	-2.96±0.55^{e}
		CHBE	-2.65±0.31^{a}	-3.12±0.51^{c}	-2.80±0.63^{d}	-2.98±0.13^{b}	-3.02±0.29^{d}	-3.19±0.37^{cd}	-2.89±0.09^{e}
	Kiwi	NC	-8.47±0.97^{b}	-8.43±0.38	-8.07±0.27^{e}	-5.81±0.48^{c}	-10.52±0.35^{f}	-9.60±1.17^{f}	-4.28±0.65^{f}
		CH	-8.86±0.94^{b}	-8.69±0.35	-8.26±0.52^{e}	-6.78±0.61^{cd}	-6.36±0.29^{e}	-5.86±0.74^{e}	-4.96±0.52^{g}
		CHBE	-8.56±0.05^{b}	-7.90±0.10	-7.65±0.66^{e}	-7.35±0.33^{d}	-6.16±0.30^{e}	-5.93±0.21^{e}	-6.35±0.12^{h}

Ns: Not subjected. Since the NC group was considered as the reference, there are no Δ*E** and Δ*C** values.

[1] Different letters in the same column (based on each analysis) indicate significant differences among the sample groups according to the Duncan's test ($P<0.05$).

Table S3. Microbial load (log CFU g^{-1}) of fruit salads during storage (NC: non-coated; CH: chitosan-coated; CHBE: bergamot juice powder extract-enriched chitosan-coated)[1].

Analysis	Sample group	Storage time (day)						
		0	3	6	9	12	15	18
Total mesophilic aerobic bacteria	NC	4.33±0.16^{a}	4.78±0.12^{a}	5.60±0.14^{a}	5.87±0.10^{a}	6.11±0.20^{a}	6.20±0.21^{a}	6.27±0.14^{a}
	CH	3.12±0.17^{b}	3.29±0.15^{b}	3.40±0.10^{b}	3.51±0.11^{b}	3.59±0.90^{b}	3.72±0.17^{b}	3.76±0.15^{b}
	CHBE	3.01±0.23^{b}	3.04±0.15^{b}	3.13±0.40^{b}	3.15±0.22^{c}	3.17±0.11^{b}	3.22±0.10^{c}	3.24±0.12^{c}
Total psychrotrophic bacteria	NC	3.26±0.13^{a}	3.50±0.07^{a}	3.87±0.17^{a}	4.62±0.15^{a}	4.87±0.10^{a}	5.05±0.11^{a}	5.18±0.15^{a}
	CH	2.96±0.10^{b}	3.00±0.13^{b}	3.09±0.15^{b}	3.21±0.20^{b}	3.59±0.14^{b}	3.70±0.11^{b}	3.81±0.18^{b}
	CHBE	2.66±0.16^{c}	2.68±0.24^{b}	2.70±0.09^{c}	2.78±0.19^{c}	3.00±0.14^{c}	3.02±0.21^{c}	3.07±0.15^{c}
Total yeast and mold	NC	3.94±0.05^{a}	4.24±0.08^{a}	4.50±0.10^{a}	5.02±0.13^{a}	5.56±0.17^{a}	6.57±0.26^{a}	7.09±0.01^{a}
	CH	3.10±0.08^{b}	3.12±0.06^{b}	3.51±0.05^{b}	3.57±0.01^{b}	3.63±0.02^{b}	3.67±0.05^{b}	3.80±0.03^{b}
	CHBE	2.70±0.01^{c}	2.76±0.06^{c}	2.80±0.01^{c}	2.88±0.01^{c}	2.93±0.04^{c}	3.08±0.07^{c}	3.11±0.04^{c}
Total *Enterobacteriaceae*	NC	3.43±0.01^{a}	3.47±0.02^{a}	3.48±0.02^{a}	3.75±0.05^{a}	3.86±0.03^{a}	3.98±0.02^{a}	4.19±0.01^{a}
	CH	2.13±0.05^{b}	2.18±0.08^{b}	2.30±0.04^{b}	2.57±0.04^{b}	2.60±0.10^{b}	2.64±0.08^{b}	2.50±0.12^{b}
	CHBE	2.07±0.01^{c}	2.09±0.13^{b}	2.11±0.07^{c}	2.15±0.04^{c}	2.24±0.05^{c}	2.29±0.02^{c}	2.30±0.10^{c}

[1] Different letters in the same column (based on each analysis) indicate significant differences among the sample groups according to the Duncan's test (*$P<0.05$*).

Table S4. Sensory scores (rating out of 5) of fruit salads during storage (NC: non-coated; CH: chitosan-coated; CHBE: bergamot juice powder extract-enriched chitosan-coated)[1].

Analysis	Sample group	Storage time (day)						
		0	3	6	9	12	15	18
Odor	NC	5.00±0.00^{a}	3.81±0.43^{b}	3.25±0.50^{b}	1.75±0.83^{b}	1.00±0.00^{c}	1.00±0.00^{c}	1.00±0.00^{c}
	CH	5.00±0.00^{a}	5.00±0.00^{a}	4.50±0.25^{a}	4.20±0.45^{a}	4.00±0.71^{b}	3.25±0.96^{b}	3.10±0.77^{b}
	CHBE	5.00±0.00^{a}	5.00±0.00^{a}	5.00±0.00^{a}	5.00±0.00^{a}	4.90±0.07^{a}	4.80±0.14^{a}	4.65±0.21^{a}
Texture	NC	5.00±0.00^{a}	4.80±0.15^{b}	4.05±0.18^{a}	3.75±1.16^{a}	2.70±0.50^{b}	2.10±0.35^{b}	1.54±0.27^{c}
	CH	5.00±0.00^{a}	5.00±0.00^{a}	4.80±0.27^{a}	4.50±0.42^{a}	4.00±0.80^{a}	3.70±0.79^{ab}	3.25±0.49^{b}
	CHBE	5.00±0.00^{a}	5.00±0.00^{a}	4.90±0.10^{a}	4.88±0.24^{a}	4.70±0.18^{a}	4.60±1.80^{a}	4.54±0.40^{a}
Appearance	NC	4.50±0.50^{a}	3.00±1.00^{b}	2.69±0.12^{b}	1.97±1.23^{b}	1.00±0.12^{c}	1.00±0.00^{c}	1.00±0.00^{c}
	CH	5.00±0.00^{a}	5.00±0.00^{a}	4.90±0.10^{a}	4.50±0.42^{a}	4.30±0.35^{b}	3.70±0.19^{b}	3.25±0.49^{b}
	CHBE	5.00±0.00^{a}	5.00±0.00^{a}	5.00±0.00^{a}	4.95±0.15^{a}	4.92±0.06^{a}	4.87±0.35^{a}	4.75±0.19^{a}
Taste	NC	5.00±0.00^{a}	3.85±0.35^{b}	1.80±1.42^{b}	Ns	Ns	Ns	Ns
	CH	4.85±0.15^{a}	4.50±0.20^{a}	4.38±0.43^{a}	4.00±0.71^{a}	3.92±0.47^{a}	3.25±0.49^{b}	3.00±0.22^{b}
	CHBE	4.95±0.05^{a}	4.70±0.10^{a}	4.61±0.21^{a}	4.52±0.37^{a}	4.30±0.26^{a}	4.08±0.26^{a}	3.74±0.34^{a}
Overall preference	NC	5.00±0.00^{a}	3.50±0.50^{b}	2.70±1.12^{b}	2.05±1.19^{b}	1.00±0.50^{c}	1.00±0.00^{c}	1.00±0.00^{c}
	CH	4.80±0.20^{a}	4.70±0.20^{a}	4.30±0.54^{a}	4.10±0.45^{a}	3.90±0.65^{b}	3.75±0.19^{b}	3.15±0.40^{b}
	CHBE	5.00±0.00^{a}	5.00±0.00^{a}	5.00±0.00^{a}	4.96±0.47^{a}	4.90±0.17^{a}	4.72±0.26^{a}	4.56±0.44^{a}

Ns: Not subjected. Fruit have not been consumed in the following periods.

[1] Different letters in the same column (based on each parameter) indicate significant differences among the sample groups according to the Duncan's test (*$P<0.05$*).
